# A phase II trial of gemcitabine plus carboplatin in advanced transitional cell carcinoma of the urothelium

**DOI:** 10.1186/1471-2407-7-98

**Published:** 2007-06-09

**Authors:** Nong Xu, Xiao Chen Zhang, Jian Ping Xiong, Wei Jia Fang, Lan Fang Yu, Jiong Qian, Ling Zhang

**Affiliations:** 1 Department of Medical Oncology, The First Affiliated Hospital, School of Medicine, Zhejiang University, 79 Qingchun Rd. 310003 Hangzhou, China; 2 Department of Oncology, The First Affiliated Hospital, Nanchang University, 17 Yongzhengwai Rd. 330006 Nanchang, China

## Abstract

**Background:**

Recent studies have demonstrated the effectiveness of cisplatin-based combinations in patients with advanced transitional cell carcinoma(TCC) of the urothelium. Concern over cisplatin toxicity instigated a search for alternative regimens. The aim of the study was to evaluate the activity and tolerability of gemcitabine plus carboplatin combination as first-line treatment in patients with advanced transitional cell carcinoma of the urothelium.

**Methods:**

Patients with advanced TCC were treated with gemcitabine 1200 mg/m^2 ^on days 1 and 8 and carboplatin area under the concentration-time curve(AUC) 5 on day 1 every 21 days.

**Results:**

Out of 41 patients, thirty-nine were evaluable for efficacy and 41 for toxicity. A median of 5 cycles (range 1–6) was administered. Overall response rate was 46.2% (95% confidence interval: 32–65%) including 10.3% complete responses and 35.9% partial responses. The median time to progression and median overall survival were 7.5 months (95% confidence interval: 6.6–8.4 months) and 13.6 months (95% confidence interval: 10.2–17.0 months), respectively. Grade 3/4 neutropenia, anemia and thrombocytopenia were observed in 36.6%, 26.8, and 24.4% of patients, respectively. Non-hematological toxicity was generally mild. Grade 3 vomiting occurred in 1 (2.4%) patients.

**Conclusion:**

The gemcitabine plus carboplatin combination is active in advanced TCC with acceptable toxicity and needs to be evaluated further and compared with other non-cisplatin-containing regimens.

**Trial registration:**

ISRCTN88259320

## Background

Transitional cell carcinoma of the bladder globally affects 356 000 men and women annually with 145 000 deaths resulting from the disease [1]. Treatment options for patients with advanced transitional cell carcinoma(TCC) of the urothelium include combination chemotherapy with methotrexate, vinblastine, doxorubicin and cisplatin (MVAC) [2,3] or gemcitabine plus cisplatin (GC) [4,5]. Both are effective regimens but have substantial cisplatin-induced toxicities. While cisplatin-based regimens comprise the mainstay of treatment in advanced TCC, many patients with this disease are elderly and often present with significant co-morbidities rendering them especially vulnerable to the toxicities associated with these current regimens. Thus, there is a considerable need to response data effective treatment for patients with advanced TCC who are not suited for cisplatin-containing chemotherapy. Carboplatin has several advantages over cisplatin in the palliative setting. Its more favourable toxicity profile and the ability to attain more predictable hematological toxicity by dosing to AUC make it a good alternative in patients with imperfect renal function. Single agent carboplatin has response rates ranging from 8–18% in advanced TCC [6-8]. Gemcitabine is a nucleoside antimetabolite that has a single-agent response rate of approximately 24–50% as both first and second-line therapy[9]. In addition, gemcitabine has a good toxicity profile and interacts synergistically with platinum [10], making it an attractive drug to use in combination with carboplatin. Data regarding the efficacy of carboplatin-containing chemotherapy are limited in patients who are fit for cisplatin-based treatment. In addition, patients in China prefer the schedule of a split dose of cisplatin on day 1 through day 3 due to less emesis. The combination of gemcitabine and carboplatin is feasible to be especially evaluated in the Chinese population.

On the basis of these considerations, we conducted a single-arm phase II study to evaluate the objective response rate and tolerability of the combination of gemcitabine and carboplatin in Chinese patients with advanced transitional cell carcinoma of the urothelium. Secondary objective was to assess the impact of this regimen on overall survival and progression-free survival.

## Methods

### Eligibility criteria

Patients with locally advanced or metastatic transitional cell carcinoma of the bladder, ureter or renal pelvis were eligible for this study. Patients were required to have histologically or cytologically proven advanced TCC and measurable disease. Prior cytotoxic treatment either in the adjuvant setting or for metastatic disease was permitted if the treatment had been completed at least six months prior to enrollment in the study. Prior radiotherapy was permitted but must have been completed at least six weeks prior to enrollment. Other eligibility criteria were: Eastern Cooperative Oncology Group(ECOG) performance status ≤ 2, a life expectancy > 3 months, age between 18 and 75 years, adequate bone marrow (absolute neutrophil count ≥ 1.5 × 10^9^/L, platelet count ≥ 100 × 10^9^/L, and hemoglobin > 10 g/dL), hepatic function (aspartate aminotransferase/alanine aminotransferase, AST/ALT) ≤ 3.0 times the upper normal limit (UNL), renal (serum creatinine ≤ 1.5 × UNL and creatnine clearance ≥ 30 ml/min based on the Calvert formula[11]) and liver (serum bilirubin ≤ 1.5 × UNL) functions, normal cardiac function, absence of second primary tumor other than non-melanoma skin cancer or in situ cervical carcinoma, no CNS involvement, no prior radiotherapy in parameter lesions, and no concurrent uncontrolled medical illness. The study was conducted in accordance with the Helsinki declaration and the guidelines on good clinical practice. In addition, the study protocol was approved by the appropriate ethical review boards and each patient provided written consent prior to study entry.

### Treatment schedule and dose adjustments

Gemcitabine 1200 mg/m^2 ^was given by intravenous infusion over 30 minutes on day I and 8 of a 21-day cycle. Carboplatin dosed to an AUC of 5 was given as an intravenous infusion over one hour on day I of a 21-day cycle. Carboplatin dosage calculation was based on the glomerular wltration rate according to the Calvert formula[11]. Patients were reviewed every three weeks for toxicity. All toxicity was recorded according to the National Cancer Institute common toxicity (NCI-CTC) criteria(version 2.0). Dose adjustments during the treatment were based on hematological and non-hematological toxicities. On day 1, if neutrophil count was < 1.5 × 10^9^/L and/or platelet count was < 100 × 10^9^/L, chemotherapy doses were delayed (for up to 2 weeks) and doses were reduced by 25% to allow recovery from hematological toxicity. On day 8, for a neutrophil count < 1.0 × 10^9^/L and/or platelets < 75 × 10^9^/L, the gemcitabine dose was omitted, and the cycle continued with one gemcitabine dose not given. Patients not recovering from hematological toxicity (neutrophil count > 1.0 × 10^9^/L and platelets > 75 × 10^9^/L) within 2 weeks were withdrawn from the trial. Doses were reduced by 25% for any grade 3 non-hematological toxicity (excluding nausea, vomiting and alopecia). Treatment was discontinued in the event of grade 4 or frequent grade 3 non-hematological toxicity. For grade 2–4 neurological toxicity, carboplatin treatment was delayed until the patient recovered to grade 1; then the dose was reduced by 25%. If no recovery to grade 1 was achieved within 3 weeks, the patient was discontinued from the trial. Blood transfusions, anti-emetics and analgesics were administered as appropriate. Patients received a maximum of six cycles unless they developed progressive disease or toxicity unacceptable to the patient.

### Baseline and treatment assessments

Pretreatment evaluation included clinical history and physical examination, automated blood cell count, biochemical profile, ECG, and computed tomography of thorax and abdomen. Blood counts were obtained twice a week; biochemical profile was repeated every 3 weeks. All measurable parameters of disease were reevaluated every 6 weeks, until the tumor progressed. Cardiac monitoring was performed at baseline with ECG repeated every cycle. Patients were evaluated for response to chemotherapy every two cycles of treatment. Responses were assessed by at least two observers, and were confirmed by an expert independent radiologist. The response evaluation criteria in solid tumors (RECIST) criteria were used to evaluate clinical response [12]. Assessment of progression-free survival (PFS) was determined by measuring the time from the date of study entry to the first date of documented progression or death from any cause. Overall survival (OS) was determined by measuring the time from study entry to time of death due to any cause or last contact. Toxicity was assessed in each treatment cycle of therapy using the NCI-CTC 2.0 criteria.

### Statistical consideration

The primary end point of this study was to estimate the overall response rate of the regimen. Secondary end points were PFS, OS and safety. The Optimal Simon two-stage phase II design was used to determine the sample size. If the results of the trial were compatible with a 50% response rate in the population under study, the combination would be further investigated; however, if the results were unable to demonstrate at least a 30% response rate in the population under study, the combination would be rejected for further investigation. Therefore, interim analysis was carried out when the first 19 assessable patients had been recruited[13]. If more than six responses were observed, 20 additional patients were to be recruited; otherwise, the study was to be terminated. If more than 16 responses were observed in the 39 patients, the regimen was considered sufficiently active with a significance level of 5% and power of 80% to be submitted for further evaluation. PFS and OS were analyzed according to the Kaplan-Meier method, and were updated to 15 June 2006. The relationship between survival and each of risk groups was analyzed by using the log-rank test. Statistical computations were performed using SPSS 11.0 for Windows procedures(SPSS Inc., Chicago, IL, USA).

## Results

### Patient characteristics

From January 2003 to June 2006, 41 patients with advanced transitional cell carcinoma(TCC) of the urothelium were entered onto this trial. Thirty-nine patients were evaluable for efficacy and 41 patients for toxicity. The pretreatment characteristics of patients are listed in Table 1. None of the patients had previously received chemotherapy for advanced disease. Two patients were excluded from the response analysis because they did not complete two cycles of chemotherapy and did not show early progression; two patients refused continuation of treatment because of personal circumstances after the first cycle.

**Table 1:** Patient characteristics

	No.	%
No. included	41	100
Median age (years)	64.5(45–75)	
Male/female	31	75.6
	10	24.4
ECOG-PS		
0	9	21.9
1	27	65.9
2	5	12.2
Primary tumour		
bladder	30	73.2
ureter	5	12.2
renal pelvis	6	14.6
Creatinine clearance (ml/min)		
≥ 30 and < 60	8	19.5
≥ 60	33	80.5
Disease status		
Locally advanced disease	17	41.5
Metastases disease	24	58.5
Lymph nodes	18	43.9
Bones	5	12.2
Lung	6	14.6
Liver	5	12.2
Multiple site involvement	6	25

### Efficacy

**Figure 1:**
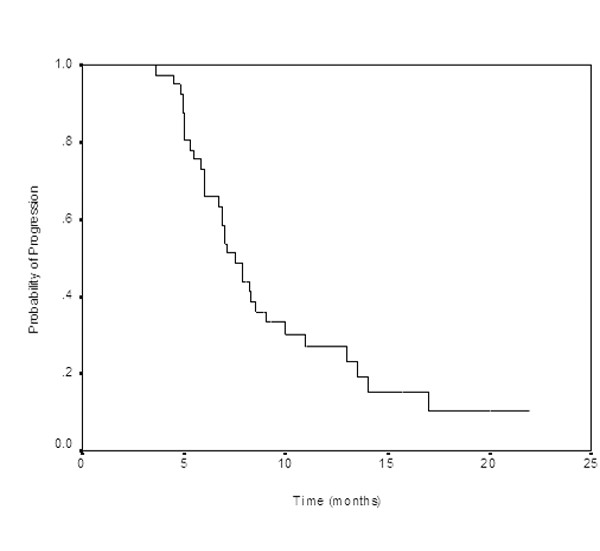
Progression-free survival for all patients.

**Figure 2:**
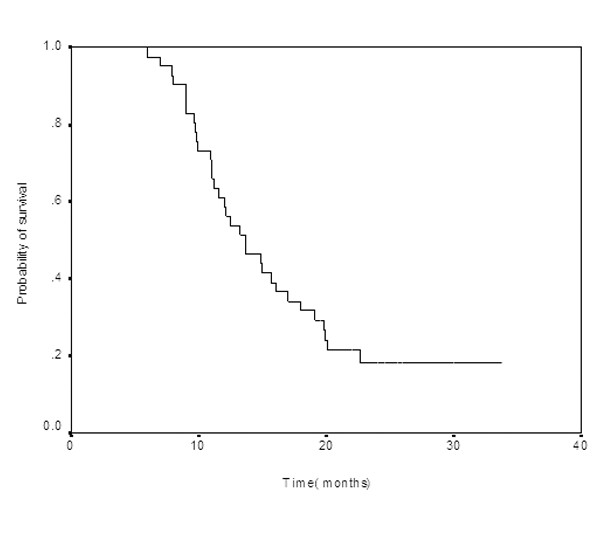
Overall survival for all patients.

Among 39 assessable patients, we observed four (10.3%) complete responses (CRs), 14 (35.9%) partial responses (PRs), for an overall response rate(ORR) of 46.2% (95% CI:32–65%). As per the intent-to-treat (ITT) analysis, the ORR was 43.9%(95% confidence interval [CI] :31–63%). Fifteen (38.4%) patients had stable disease and 6 (15.4%) had progressive disease. Median PFS was 7.5 months (95% CI: 6.6–8.4 months) (Figure 1) and median OS was 13.6 months (95% CI: 10.2–17.0 months) (Figure 2). One- and 2-year survivals were 58.5% and 18.3%, respectively. Thirty-three patients had died at the time of the present evaluation. The median follow-up was 19.2 months (rage: 10.8–35 months).

Based on Bellmunt et al's report [14], the ECOG performance status and the presence of visceral metastases are two pretreatment risk factors. In the current study the number of patients with zero-risk (ECOG performance status 0 and no visceral metastasis), one-risk(performance status > 0 or visceral metastasis), and two-risk factors(performance status > 0 and visceral metastasis) was 9(23.1%), 19 (48.7%), and 11(28.2%), respectively. Patients with no risk factors had a median survival time of 22.6 months (95% CI, 16.0–29.2 months), patients with one risk factor had a median survival time of 13.6 months (95% CI, 11.9–15.3 months), and patients with the two risk factors had a median survival time of 9.6 months (95% CI, 7.7–11.5 months). There was a significant difference in survival profiles of the three risk groups (p < 0.0000). One patient had PR, 2 had SD and 2 had PD in five patients with bone metastases. Patients with bone metastases had a median survival time of 9.8 months (95% CI, 8.1–11.5 months), patients without bone metastases had a median survival time of 13.6 months (95% CI, 10.0–17.2 months)(p = 0.0428).

A total of 199 chemotherapy cycles were administered, with a median of 5 cycles per patient (range 1–6), and 2(4.9%) patients received 1 cycles, 3(9.8%) 3 cycles, 9(21.9%) 4 cycles, 7 (17.1%) 5 cycles and 19 (46.3%) 6 cycles. The planned dose intensity was 800 mg/m^2 ^/week for gemcitabine and 1.67 AUC/week for carboplatin. Dose intensity for all 41 patients was 731 mg/m^2 ^/week for gemcitabine and 1.55 AUC/week for carboplatin, with 91.4% of the planned gemcitabine dose and 92.8% of the planned carboplatin dose delivered.

### Toxicity

A total of 199 treatment cycles were administered, with an average of 5 cycles per patient (range, 1–6 cycles). Thirty-nine patients received three cycles and 19 patients received at least six cycles. In all, 6 patients (15.4%) discontinued due to disease progression, 10(24.4%) due to toxicity: 6(14.6%) hematological toxicity and 2 (4.9%) due to other toxicity, 6(14.6%) due to a decision of the patient. The frequencies of hematological and non-hematological toxicities are shown in Table 2. Hematological toxicities were the main side effects. Grade 3/4 neutropenia occurred in 15(36.6%) patients. However, only 2 of these patients had neutropenia fever. Grade 3/4 thrombocytopenia was observed in 10 (24.4%) patients. Grade 3/4 anemia was reported in 11(26.8%) patients. Patients required platelet transfusion in 18 cycles (4.4%), and hematopoietic growth factors support care in 43(21.6%) cycles. No bleeding episodes were recorded. Non-hematological toxicity was generally mild. The most of toxicities were grade 1 to 2, with only one (2.4%) patient had grade 3 vomiting. Grade 1/2 Fatigue occurred in 7 (17.1%) patients. No serious adverse events were reported during the study and none of the patients died from the toxicity. Although the protocol specified 21 days between cycles, 163 cycles (81.9%) received all treatment cycles within the protocol 21-day period. 25 cycles (12.6%) were delayed fewer than seven days, 11 cycles (5.5%) were delayed more than seven days. Of these, 28 cycles (14.1%) were delayed for toxicity reasons.

**Table 2:** Hematological and non-hematological toxicities according to NCI-CTC (n = 41)

	Grade 1(%)	Grade 2(%)	Grade 3(%)	Grade 4(%)
Leucocytopenia	12(29.3)	15(36.6)	6(14.6)	2(4.9)
Neutropenia	6(14.6)	14(34.1)	11(26.8)	4(9.7)
Febrile neutropenia	2(4.9)	0	0	0
Anemia	4(9.8)	16(39.0)	7(17.1)	4(9.7)
Thrombocytopenia	4(9.8)	5(12.2)	6(14.6)	4(9.7)
Nausea	15(36.6)	6(14.6)	0	0
Vomiting	7(17.1)	5(12.2)	1(2.4)	0
Diarrhea	2(4.9)	0	0	0
Increased ALT	4(9.8)	1(2.4)	0	0
Increased creatinine	4(9.8)	0	0	0
Alopecia	5(12.2)	2(4.9)	NA	NA
Neurological toxicitity	1(2.4)	0	0	0
Mucositis	2(4.9)	0	0	0
Fatigue	5(12.2)	2(4.9)	0	0

ALT, alanine aminotransferase;

NA, not appolicable;

NCI-CTC, National Cancer Institute Toxicity Critrria

## Discussion

Treatment of patients with advanced transitional cell carcinoma (TCC) of the urothelium is difficult. Advanced age, concomitant diseases, poor performance status, frequent deterioration of renal function, and frequent palliative treatment underscore the need to search for a treatment scheme with a good efficacy/toxicity profile. Cisplatin-based combination chemotherapy (MVAC and GC regimen) represents the most effective treatment for patients with advanced TCC. Nevertheless, at least one-third of patients with inoperable bladder cancer are unfit to receive cisplatin-based chemotherapy. In recent years, the development of new drugs, such as paclitaxel, docetaxel, and gemcitabine, has begun to change a somewhat discouraging prospect. Because of the known activity of gemcitabine and its synergy with platinum agents, the substitution of carboplatin for cisplatin in this combination is a promising alternative in the treatment of advanced TCC patients. In addition, carboplatin can be given safely to most patients, particularly those with moderate renal insufficiency. The present study suggests that the combination of gemcitabine plus carboplatin is an effective and well-tolerated regimen in the treatment of advanced TCC patients in the Chinese population. This combination regimen demonstrated promising efficacy with a tumor response rate of 46.2%, a median PFS of 7.5 months and a median overall survival of 13.6 months. And this is comparable to the results reported from studies regarding the combination of gemcitabine plus carboplatin [15-22].

Gemcitabine was initially evaluated in an Italian phase I study conducted in 15 patients with metastatic bladder cancer [23]. The doses ranged from 875 to 1370 mg/m^2^. One complete response and 2 partial responses were seen in 14 previously treated patients and 1 partial response was observed in a chemotherapy-naive patient. The overall response rate was 27%. In two phase II trials in previously treated patients, a response rate of 28% and 50% was reported [24,25]. Two trials evaluating gemcitabine in previously untreated patients confirmed the high activity of this agent. Stadler et al [26] treated 40 patients with gemcitabine 1200 mg/m^2 ^weekly times three, repeated every 28 days, and reported an overall response rate of 28%. Additionally, Moore et al confirmed in 37 non treated patients an overall response rate of 24.3% [27]. Gemcitabine has been studied in combination with carboplatin in different studies. Before our study, eight additional phase II trials were conducted to evaluate the activity and toxicity of the GC combination in advanced TCC. The first small study conducted by Carles et al and included 17 patients with impaired renal function (creatinine clearance: 21–55 ml/min) [15]. Patients received gemcitabine 1000 mg/m^2 ^on days 1 and 8 of a 21-d schedule plus carboplatin AUC 5 on day 1. 56% of patients obtained an objective response, with a median survival time of 10 months. Three small trials used the same schedule and doses as those of the Carles trial and demonstrated response rates in the range of 44–61% with the GC combination [16-18]. In addition four larger phase II studies, Nogue-Aliguer et al reported their results in 41 patients, of whom some, but not all, had unfavorable characteristics (creatinine clearance less than 60 ml/min in 54% of patients and Karnofsky performance status of 70 or less in 37%). The overall response rate was 56.1%, with progression-free survival (PFS) of 7.2 months and overall survival of 10.1 months[19]. A study conducted by Linardou et al with similar characteristics to Nogue-Aliguer's study showed a 36% overall response rate (ORR) with a time to progression(TTP) of 4.8 months and an OS of 7.2 months in 56 patients[20]. The other two studies included the most of patients with favorable characteristics (creatinine clearance more than 60 ml/min and good performance status) [21,22]. Patient received gemcitabine 1000–1200 mg/m^2 ^on days 1 and 8 of a 21-d schedule plus carboplatin AUC 5 on day 1. The results in 50 and 60 patients showed a 56% and 38% ORR with a median PFS/TTP of 11 and 7.6 months and a median OS of 11.3 and 16.3 months, respectively. Table 3 summarises the results of our study in comparison of the published phase II studies using gemcitabine and carboplatin combination as first-line chemotherapy in advanced TCC patients. The promising activity of taxanes led to the development of new triplets by the addition of this agent to the gemcitabine-carboplatin combination. Hussain et al. conducted a phase II trial evaluating the efficacy of the combination paclitaxel-carboplatin-gemcitabine in patients with advanced TCC [28]. Most of the 49 patients who were enrolled would have been eligible for cisplatin-based chemotherapy. The ORR was 68%, with a CR rate of 32% and a median survival of 14.7 months. In a similarly designed study, Hainsworth et al. failed to duplicate these results[29]: lower RR (43%), CR rate (12%) and median survival (11 months) in 60 patients with similar prognostic features were reported. DiPaola et al. investigated a novel schedule of gemcitabine, paclitaxel, and carboplatin[30]. Administration of gemcitabine and paclitaxel followed by carboplatin was well tolerated and clinically active. Eighteen patients had advanced urothelial cancer on trial and 15 had at least two cycles of therapy, of which two had a CR and 6 had a PR. Hoshi et al. have reported the efficacy of two combined chemotherapy regimens in previously treated patients with a platinum-based regimen[31]: gemcitabine plus carboplatin(GCa), and gemcitabine, docetaxel, and carboplatin(GDCa). The ORR was 46%(7/15) and 67%(6/9) in GCa and GDCa regimens, respectively. Five of the 8 (63%) GCa-refractory patients responded to GDCa therapy.

**Table 3:** comparison of the published phase II studies using gemcitabine and carboplatin combination as first-line chemotherapy in advanced TCC patients

Study	N	RR (%)	TTP/PFS*(mo)	OS(mo)	Neutrop-enia G3/4%	Anemia G3/4%	Thromboc-ytopenia G3/4%	CrCl < 60 or < 50* (%)	Median Age(y)	PS ≥ 2, ≤ 70%* (%)	visceral mets (%)
Carles^15^	17	56.3	NR	10	24	18	18	100	69	41*	29
Shannon^16^	17	58.8	4.6	10.5	70	18	47	77	69	29	59
Bellmunt^17^	16	44	NR	NR	56	50	63	100	68	13	31
Hoschke^18^	23	60.8	7.8	15.4	26*	30	48	52	68	21	43
Nogue-Aliguer^19^	41	56.1	7.2*	10.1	63	54	32	54	66	37*	20
Linardou^20^	56	36	4.8	7.2	28	18	17	68*	75	46	39
Olivares^21^	50	56	11*	11.3	42	18	20	NR	69	18*	18
Bamias^22^	60	38.3	7.6	16.3	52	18	23	22*	69	13	52
this study	41	46.2	7.5*	13.6	37	27	24	20	67	15	27

NR, Not reported

The toxicity profile in this study is similar to that of previous reports [15-22]. Grade 3/4 neutropenia, anemia and thrombocytopenia were observed in 36.6%, 26.8% and 24.4% of patients, respectively. Non-hematological toxicity was generally mild. Compared with MVAC, Grade 3/4 neutropenia, at a rate of approximately 37%, was lower in our study compared with the reported 67–82% in the most randomized studies [4,5,32]. A pretreatment performance status > 0 (or KPS < 80%) and the presence of visceral metastasis have a profound impact on survival when using both the M-VAC regimen and new drug-based regimens, including paclitaxel and gentacibine [14,33]. Similar to the results of those trials conducted by Bellmunt et al. and Bajorin et al., patients with no risk factors had a longer survival than patients with one or two risk factor(s) in this study. In addition, patients with bone metastases had a poor survival. Four of five patients with both visceral metastasis and bone metastases had no response. Only one patient with bone and lymph node metastases had PR. Chemotherapy combined with bisphosphonates and radiation therapy can be selected based on the location and extent of bone metastases. Bone metastases of urothelial cancer is really difficult to handle. A new strategy needs to be developed. In general, gemcitabine and carboplatin combination demonstrated promising efficacy with a ORR of 36–61%, a median TTP/PFS of 4.6–7.8 months and a median overall survival of 7.2–16.3 months, in spite of differences in baseline characteristics (the frequencies of advanced age, impaired renal function, poor performance status and visceral metastasis) and treatment after the completion of chemotherapy.

In patients unfit for cisplatin-based therapy carboplatin is usually substituted for cisplatin in everyday practice to produce less nephrotoxic and more tolerable regimes. But the question whether carboplatin can be substituted for cisplatin in patients with unimpaired renal function without compromising efficacy remained unsolved during the MVAC era. Two single-institution randomized phase II studies have been reported [34,35]. Petrioli et al treated 55 patients with methotrexate, vinblastine, epirubicin, and either cisplatin or carboplatin. Lower levels of gastrointestinal, renal, neurologic, and otologic toxicity were seen in the carboplatin arm. Results regarding efficacy suggested a better efficacy for the cisplatin-based regimen when considering the overall response rate (71% in the cisplatin arm vs. 41% in the carboplatin arm) and the median survival (13 vs. 9.5 months)[34]. Similar trends were reported by Bellmunt et al in treating 47 patients with MVAC or a combination of methotrexate, vinblastine, and carboplatin. MVACa was considered more toxic but also more active than the carboplatin-based regimen because overall response rates were 52% and 39% and median survivals were 16 and 9 months, respectively [35]. More recently, ECOG conducted a phase III trial comparing MVAC with a combination of paclitaxel and carboplatin. It was closed early due to poor accrual after enrolling 85 patients. No differences were observed in measured quality of life and median survival [32]. Because all these trials were clearly underpowered, the role of carboplatin in patients with advanced TCC could not be precisely defined. Based on this background, Doglietti et al. recently conducted a randomized phase II study comparing the toxicity and efficacy of gemcitabine and cisplatin (GC) or gemcitabine and carboplatin (GCa) in patients with unimpaired renal function [36]. At the end, no differences were observed in the overall toxicity profile and any parameter of toxicity. Overall response rates were 49% and 40% and median survivals were 12.8 and 9.8 months for GC and GCa, respectively. Are these results likely to have an impact on our daily practice? Data regarding the efficacy of the combination of gemcitabine and carboplatin is still limited in 'fit-for-cisplatin' patients, particularly in Chinese patients who prefer cisplatin-free combination. Only a randomized phase III trial could have precisely defined the role of carboplatin in patients fit or unfit for cisplatin-based combination chemotherapy. In addition, the integration of the new active agents in two, three cisplatin-free combinations, such as taxanes, oxaliplatin, and premetrexed, should be tested in randomized trials. The development of new cytotoxic agents or targeted therapies is urgently required to improve outcomes.

## Conclusion

The combination of gemcitabin and carboplatin in patients with advanced TCC exhibits an acceptable toxicity profile and produces response rates at least comparable to cisplatin based combinations. It needs to be evaluated further and compared with other non-cisplatin-based regimen, above all the most of patients in China prefer to receive cisplatin-free combination.

## Competing interests

The author(s) declare that they have no competing interests.

## Authors' contributions

NX and JPX conceived of the study, participated in its design and coordination and had an important role in the preparation of the manuscript. WJF and XCZ participated in the design and conduction of the study. LFY and LZ participated in the database compilation and conduction of the study. JQ supervised all the steps of the study conduction. All authors read and approved the final manuscript.

## Pre-publication history

The pre-publication history for this paper can be accessed here:


